# Pseudogene AKR1B10P1 enhances tumorigenicity and regulates epithelial‐mesenchymal transition in hepatocellular carcinoma via stabilizing SOX4

**DOI:** 10.1111/jcmm.15790

**Published:** 2020-09-13

**Authors:** Fengjie Hao, Xiaochun Fei, Xinping Ren, Joanna Xi Xiao, Yongjun Chen, Junqing Wang

**Affiliations:** ^1^ Department of General Surgery Hepatobiliary Surgery Ruijin Hospital Shanghai Jiao Tong University School of Medicine Shanghai People’s Republic of China; ^2^ Department of Pathology Ruijin Hospital Shanghai Jiao Tong University School of Medicine Shanghai People’s Republic of China; ^3^ Department of Ultrasound Ruijin Hospital Shanghai Jiao Tong University School of Medicine Shanghai People’s Republic of China; ^4^ Shanghai Institute of Digestive Surgery Ruijin Hospital Shanghai Jiao Tong University School of Medicine Shanghai People’s Republic of China

**Keywords:** AKR1B10P1, hepatocellular carcinoma, metastasis, miR‐138, SOX4

## Abstract

Pseudogenes exert potential functions in tumorigenicity and tumour process in human beings. In our previous research on oncogene AKR1B10 in hepatocellular carcinoma (HCC), its pseudogene, AKR1B10P1, was preliminarily noticed being anomalistic transcribed, whereas whether AKR1B10P1 plays any specific function in HCC is poorly understood. By using shRNA transfection and lentiviral infection, we regulated the expression of ARK1B10P1 transcript and the relative targets in two ways. As we discovered, pathological transcription of AKR1B10P1 in HCC cells significantly promotes cell growth and motility either in *vitro* or in *vivo*. AKR1B10P1 was correlated with relatively dismal features of HCC. The epithelial‐mesenchymal transition (EMT) was enhanced by up‐regulating AKR1B10P1. And, a potential sequence of AKR1B10P1 transcript was discovered directly interacting with miR‐138. SOX4, a pivotal promotor of EMT, was validated as the down‐streaming target of miR‐138. Mechanistically, degradation of SOX4 mRNA induced by miR‐138 was effectively abrogated by AKR1B10P1. In conclusion, pseudogene AKR1B10P1 exerts stabilizing effect on SOX4 in HCC, associated EMT process, by directly sponging miR‐138, which post‐transcriptionally modulates SOX4’s regulating gene.

## 1 INTRODUCTION

Hepatocellular carcinoma (HCC) is one of the most commonly occurring malignancies result in high mortality.^1^, ^2^ Despite the improvements on HCC treatment, the outcome and overall survival rate of the patients remain dismal.^3^, ^4^ Thus, precise prediction of patients’ prognosis and more effective therapeutic approach on HCC raise us new challenge and necessity.

Long non‐coding RNAs (LncRNAs), composed with over 200 nucleotides, constitute the main component of non‐coding RNAs, and exert complex biological functions intracellular through various mechanisms.^5^, ^6^ As acknowledged, pseudogenes are particularly generated from duplication of parental protein coding gene along with a variety of acquired mutation, lacking of the promoter, premature stop condon or frame‐shift mutation.^7^, ^8^ The recent research has demonstrated that pseudogenes could be transcribed. The transcriptional products of pseudogenes could exert different functions through multiple approaches in the form of lncRNAs, such as gene expression modulation through miRNA decoy effects. And either tumour suppressive or oncogenic functions have been observed concerning with pseudogenes.^9^, ^10^, ^11^ For instance, the first classic tumour suppressive pseudogene, PTENP1, was discovered regulating the expression of the parental gene PTEN through binding to miR‐17, miR‐19 and miR‐21, and consequentially suppressing human malignancies, such as clear cell renal carcinoma, oral squamous carcinoma and gastric cancer.^12^, ^13^ On the contrary, pseudogenes KRASP1 and BRAFP1 protect the expression of their parental genes through competitive endogenous RNA (ceRNA) effects, and respectively activate the oncogenic MAPK pathway.^14^ However, the functions of pseudogenes in HCC are still obscured.

In our previous study, Aldo‐Keto Reductase Family 1 Member B10 (AKR1B10) was discovered highly expressed in multiple HCC cells and remarkably promotes cell growth.^15^ AKR1B10 pseudogene 1 (AKR1B10P1) is the isoform pseudogene of AKR1B10, exhibiting almost no transcriptional profile in normal hepatocytes. As we observed, AKR1B10P1 emerges a positively correlated expression compatible with its parental gene in HCC cells.

In this study, we discovered that AKR1B10P1 is correlated with dismal HCC clinicopathological features. Anomalous transcription of AKR1B10P1 significantly promotes HCC cell growth and enhances tumour metastasis through activating the epithelial‐mesenchymal transition (EMT). Sex‐determining region Y‐related high‐mobility group box 4 (SOX4) has been reported and acknowledged as a pivotal promotor of EMT process. As we found out, the expression of SOX4 was consequentially modulated along with AKR1B10P1. And we noticed a sequence of AKR1B10P1 transcription directly interacting with miR‐138, which is an up‐streaming regulator of SOX4, in the way of molecular sponges. Mechanistically, the degradation of SOX4 mRNA induced by miR‐138 was effectively abrogated by AKR1B10P1. Our findings above indicate that AKR1B10P1 might be a valuable molecular indicator and target for HCC diagnosis and therapeutic treatment.

## 2 MATERIALS AND METHODS

### 2.1 Cell culture

HCC cell lines Hep3B, HepG2 and Hu7u were purchased from Shanghai Institutes for Biological Sciences, Chinese Academy of Science (Shanghai, China), and the normal human hepatic cell line L02 was purchased as control. All cells were cultured in RPMI 1640 supplemented with 10% heat‐inactivated foetal bovine serum (FBS), incubated at an environment temperature of 37℃, with 100 µg/ml streptomycin and 100 U/ml penicillin in a humidified cell and an atmosphere of 5% CO_2_.

### 2.2 Clinical specimens

Tumour specimens of 87 patients diagnosed with HCC pathologically were collected paired with the adjacent non‐cancerous tissues performed radical resection without preoperative therapy during 2014 to 2017, at the Department of Hepatobiliary Surgery, Ruijin Hospital, Shanghai Jiao Tong University School of Medicine. Informed consent was obtained, and the study was approved by the Ethics Committee of Ruijin Hospital, Shanghai Jiaotong University School of Medicine. Clinicopathological features of the patients including gender, age, tumour size, number of lesions and grades were collected.

### 2.3 RT‐QPCR assay, western blot analysis and immunohistochemistry assay

RNA isolation from tissues and cells were carried out according to the instruction of TRIzol reagent (Invitrogen, USA). The first‐strand cDNA was synthesized using High‐Capacity cDNA Reverse Transcription Kit (ABI, USA). RT primers of the mRNAs were synthesized by Sangon Biotech Company (Shanghai, China) (Table S1). Real‐time quantitative polymerase chain reaction (RT‐qPCR) was implemented following the TaqMan Gene Expression Assays protocol (ABI, USA) according to the methods described by Qiu, et. al.^16^

Antibodies against SOX4 and the EMT‐related proteins (N‐cadherin, vimentin, E‐cadherin) were purchased (Abcam, USA) and applied following the manufactory instruction. The Western blot analysis and immunohistochemistry assay were performed as the methods we previously described.^15^ The protein expression levels detected by IHC were assigned to two experienced pathologists for blind examination and were then set into two groups as staining intensity graded subjectively: no to low staining (0～1+) and moderate to high staining (2+～3+).

### 2.4 Plasmid construction and transfection

Hep3B cells in exponential phase were prepared and transfected with shRNA suppressing AKR1B10P1 transcript through pGU6/Neo vectors (GenePharma, Shanghai, China) along with the construction of the control cells. Transfected cells were selected using a medium mixed with G418 (Santa Cruz Biotechnology, Inc; 400 μg/ml). The lentiviral vector pWPXL (Addgene, Cambridge, USA) was introduced for ectopic expressing SOX4 (pWPXL‐SOX4) to rescue the phenotype induced by AKR1B10P1 depletion, and the pWPXL‐Null was used as negative control. Hep3B cells overexpressing miR‐138 (Hep3B/miR‐138) were constructed through the mimic method similar to the description in our previous study, followed by dual‐luciferase reporter assay, respectively, in pWPXL‐SOX4 or pWPXL‐Null‐treated cells, and the negative control ones were set (NigmiR). Relative methods were referred to the literatures of Wang, et al^17^ and Lin, et al.^18^

Additionally, we conducted the same treatment on HepG2 cells and carried out related experiments in vitro, presented in Figure S5.

### 2.5 Cell proliferation assay and cell cycle analysis

Hep3B cells (1 × 10^6^) stably transfected were cultured in 96‐well microtitre plates in triplicate and incubated at 37°C with an atmosphere of 5% CO_2_ for 5 days. Microplate computer software (Bio‐Rad Laboratories, Inc, Hercules, CA, USA) was used for measuring the OD following the Cell Counting Kit‐8 (CCK‐8) assay kit protocol (Dojindo, Tokyo, Japan). The cell proliferation curves were plotted. Methods above refer to our previous research.^19^ The aforementioned cells were treated in steps with ethanol fixation, RNase A treatment and propidium iodide staining. Flow cytometry detection was conducted using FACSCalibur (Becton‐Dickinson, Franklin Lakes, NJ, USA). Cell populations at the G0/G1, S and G2/M phases were quantified through ModFit software (Becton‐Dickinson). Cell debris and fixation artefacts were excluded.

### 2.6 Cell apoptosis analysis

Cell apoptosis rate calculation was conducted using PE‐Annexin V Apoptosis Detection Kit I (BD Pharmingen, USA) according to the product instructions. Stable transfected Hep3B cells were resuspended in 1 × Binding Buffer (1 × 10^6^ cells/ml). 5 μl of FITC and 5 μl of PI were added into 100 μl of cell suspension, followed by 15 minutes of incubation in darkness, and then, 400 μl × Binding Buffer was added. The analysis of apoptosis by flow cytometry (Becton‐Dickinson, USA) was conducted. Both Annexin V‐FITC‐positive and PE‐negative cells were considered as apoptotic. The relative methods refer to the protocol by Crowley, et al.^20^

### 2.7 Cell migration and invasion assay

The cell invasion and migration capacity of were analysed using the QCMTM 24‐Well Colorimetric Cell Migration or Invasion Assay Kit (Millipore, USA) according to the methods we previously mentioned.^21^ 3 × 10^4^ stable transfected cells in 300 ml serum‐free medium were added to the upper chamber, and 10% FBS‐containing medium was used as chemoattractant in the lower chamber. ECMatrixTM was pre‐coated to the upper chamber for invasion assay, and cells on the bottom of the membrane were stained and checked after 24 hours for migration or 48 hours for invasion.

### 2.8 Mouse liver orthotopic transplantation model and tumorigenicity assay

Four‐ to five‐week‐old male BALB/c nude mice (Institute of Zoology Chinese Academy of Sciences) were housed in the Animal Laboratory Unit, Shanghai Jiao Tong University School of Medicine, China, within the pathogen‐free environment. All experiments were performed in accordance with the guidelines of the Shanghai Medical Experimental Animal Care Commission and also refer to the relative recommended protocols.^22^ HCC cells were suspended in 25 µl serum‐free DMEM mixed with 25 µl Matrigel (1:1, v/v) every 1 × 10^6^ cell. Cells above were orthotopically injected into hepatic lobes of each mouse. All mice were killed in 6 weeks post‐injection. And the diameter and weight of all the xenografted livers were measured. For histological analysis, liver and lung from mice were collected, stained with haematoxylin and eosin (HE), and detected.

### 2.9 Immunofluorescence assay

Immunofluorescence assay was conducted on the basis of the regular protocol.^23^ Cells for immunofluorescence seeded on glass coverslips in six‐well plates were fixed with 4% formaldehyde solution and permeabilized with 0.5% Triton X‐100/phosphate‐buffered saline (PBS). The cells then were blocked with 5% bovine serum albumin/PBS for 1 hour and then were incubated overnight with primary antibodies (antibodies against E‐cadherin, vimentin and N‐cadherin) at 4°C. Treated cells were incubated with fluorescent‐dye conjugated secondary antibody (Invitrogen) for 1 hour, followed by DAPI staining. Images were graphed under an inverted fluorescence microscope.

### 2.10 Dual‐luciferase reporter assay

MiR‐138 was predicted potentially interacting with AKR1B10P1 transcript through an online tool of dreamBase (http://rna.sysu.edu.cn/dreamBase/).^24^ On the other hand, miR‐138 was regarded as the up‐streaming regulator of SOX4, predicted through microcosm (http://mirecords.biolead.org). Either the mRNA of AKR1B10P1 containing the putative miR‐138 binding site or the 3’‐untranslated region (3’‐UTR) of SOX4 mRNA was intercepted, set along with the mutated ones (Table S2). Sequences above were cloned into pMIR‐REPORT luciferase vectors (Promega, Madison, WI, USA), containing Firefly luciferase, and pRL‐TK vectors containing Renilla luciferase used as control. The vectors were co‐transfected into Hep3B cells overexpressing miR‐138 and the control ones. The luciferase activity was measured by using Dual‐Glo Luciferase assay system (Promega) 48 hours post‐transfection, according to the protocol we reported.^25^

### 2.11 Statistical analysis

Statistical analysis was carried out by using SPSS 20.0. *P*‐values were calculated using an unpaired Student's *t* test and Fisher's exact test. Differences were considered statistically significant at *P*‐values < 0.05.

## 3 RESULTS

### 3.1 Pseudogene AKR1B10P1 is transcribed in HCC cell lines and tissues

AKR1B10 is the parental gene of pseudogene AKR1B10P1. We had validated highly expression of AKR1B10 in both HCC tumour cells and specimens (Figure S1). AKR1B10P1 is a pseudogene barely transcribed in most of the human tissues. However, along with the high expression of the parental AKR1B10, AKR1B10P1 was detected highly expressed in multiple human malignancies, such as cholangiocarcinoma, lung squamous cell carcinoma and HCC (Figure S2).

**FIGURE 1:**
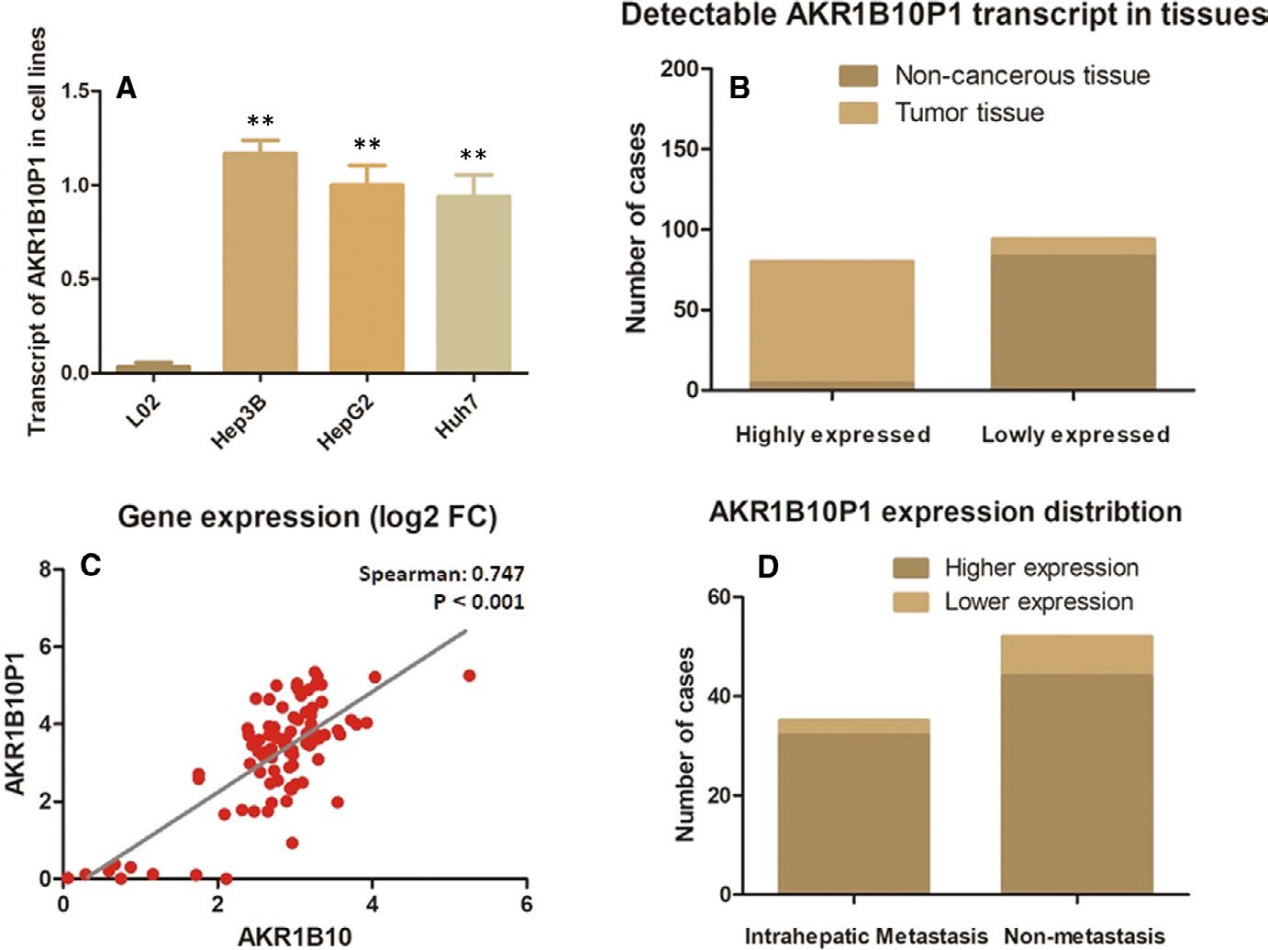
Pseudogene AKR1B10P1 is transcribed in HCC cell lines and tissues (A) RT‐qPCR assay demonstrated an obvious detectable pseudogene AKR1B10P1 transcription in HCC cell lines, while barely no expression in the control L0 cells (**P ＜ 0.01). B, AKR1B10P1 was remarkably transcribed in 87.4% of the HCC tumour tissues (76/87); only 4.6% (4/87) cases present low‐level AKR1B10P1 transcript in non‐cancerous tissues. C, The expression of AKR1B10P1 was positively correlated with its parental gene AKR1B10 in tumour tissues (Spearman *R* = 0.747, *P* < .01). D, Intra‐hepatic metastasis was detected in the 35 cases, and 91.43% cases (32/35) presented relatively higher AKR1B10P1

In our study, AKR1B1P1 was discovered highly expressed in three HCC cell lines (Hep3B, HepG2 and Hu7u), especially in Hep3B cells (Figure 1A). Simultaneously, high expression of AKR1B10P1 RNA was observed in HCC samples compared with the adjacent non‐cancerous liver tissues. As shown in Figure 1B, 87.4% (76/87) HCC specimens were remarkable expressing AKR1B10P1, positively correlated with its parental gene in tumour tissues (Spearman *R* = 0.747, *P *＜ 0.01) (Figure 1C). As for the non‐cancerous tissues, only 4.6% (4/87) cases present low detectable AKR1B10P1 transcript. Interestingly, in the 35 cases diagnosed with intra‐hepatic metastasis, 91.43% cases (32/35) presented relatively higher AKR1B10P1 expression (Figure 1D). Thus, AKR1B10P1 is supposed to relate to HCC metastasis.

### 3.2 The activation of AKR1B10P1 transcription is correlated with the HCC patients’ clinicopathologic features

The clinicopathologic features of these 87 HCC cases were analysed statistically. According to Table 1, there was no significant correlation between AKR1B10P1 and the patient's age, gender, virus control status or venous invasion were taken under consideration. However, the activation of AKR1B10P1 transcription in HCC tissues inclined to associate with larger HCC tumour size (*P *＜ 0.05), more frequency of advanced TNM stages (*P *＜ 0.05), higher serum Alpha‐fetoprotein (AFP) quantity (*P *＜ 0.01), incidence of tumour microsatellite formation (*P *＜ 0.01) and liver cirrhosis (*P *＜ 0.05).

**TABLE 1:** Correlation between AKR1B10P1 transcript and clinicopathologic features in 87 HCC specimens

Clinicopathologic parameters	AKR1B10P1 expression		P*
	Low (n = 11)	High (n = 76)	
Age (years)			
≤50	8	38	0.205
＞50	3	38	
Gender			
Male	7	41	0.748
Female	4	35	
Diameter (cm)			
≤5	9	34	0.025
＞5	2	43	
TNM stage			
I～II	8	28	0.045
III～IV	3	48	
Tumour encapsulation			
Absent	6	30	0.514
Present	5	46	
Tumour microsatellite formation			
Absent	9	28	0.007
Present	2	48	
Venous invasion			
No	6	22	0.164
Yes	5	54	
HBsAg			
Negative	3	8	0.141
Positive	8	68	
AFP(ng/ml)			
≤400	10	10	＜0.01
＞400	1	66	
Cirrhosis			
Absent	5	5	0.023
Present	6	71	

Note

AKR1B10P1 transcript level associated with clinicopathologic features in 87 HCC patients, including age, gender, tumour size, tumour stage (AJCC), tumour encapsulation, tumour microsatellite formation, vein invasion, HBsAg status, AFP level and liver cirrhosis. Statistically significance was assessed by Fisher's exact text.

### 3.3 AKR1B10P1 depletion suppresses cell proliferation in Hep3B cells and arrests the cell cycles

**FIGURE 2:**
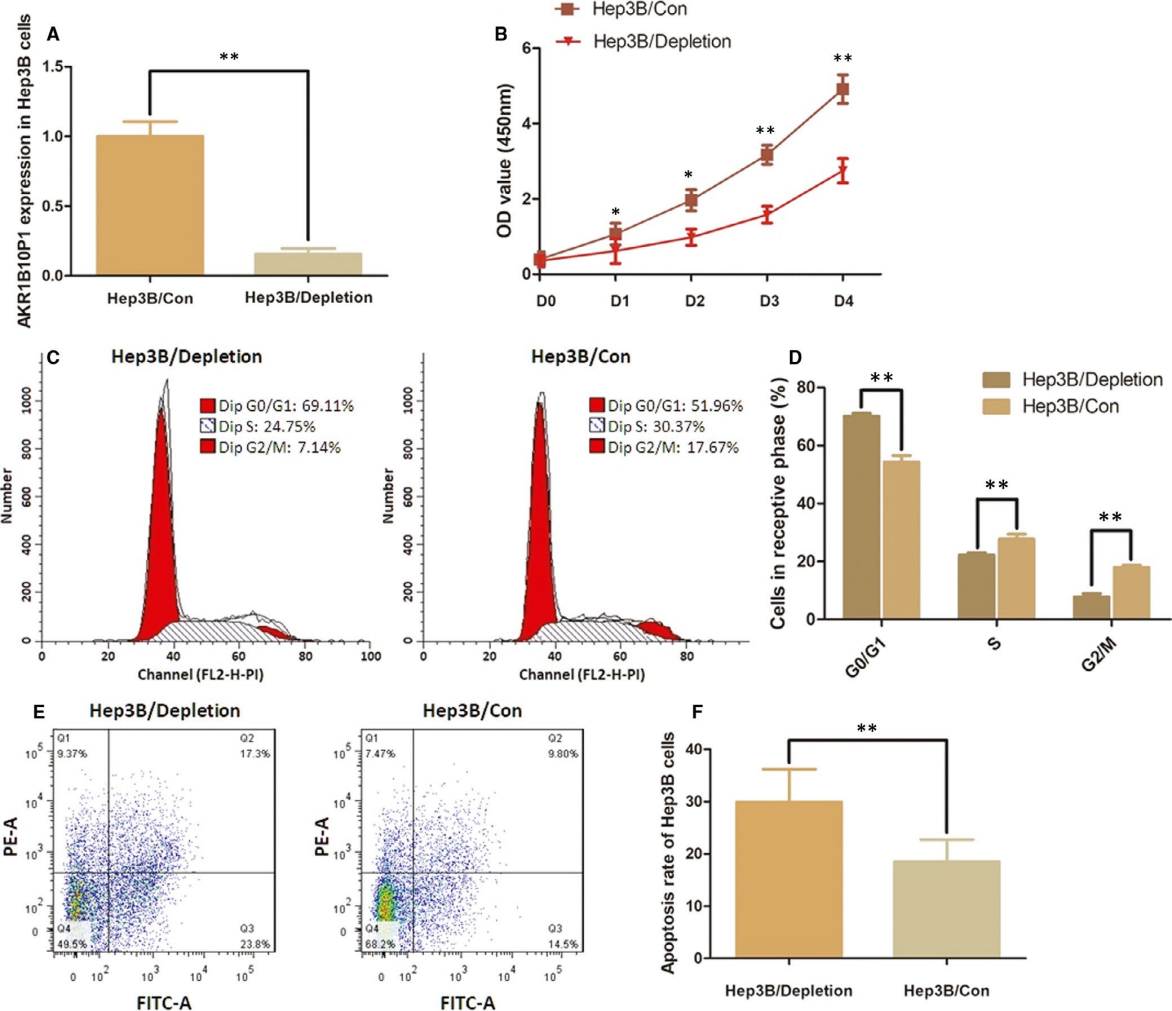
Depletion of AKR1B10P1 impairs the cell growth and promotes cell apoptosis in Hep3B cells. A, AKR1B10P1 depletion was conducted in Hep3B cells by using shRNA transfection. RT‐qPCR assay indicated a significant defection of AKR1B10P1 expression in the treated cells (**P < .01). B, CCK8 assay was applied for detection of effect of AKR1B10P1 on cell proliferation. The Hep3B cell proliferation was significantly impaired by depleting AKR1B10P1 (*P < .05, **P < .01). C and D, Flow cytometry was used for analysing the effect of AKR1B10P1 on cell cycle. As shown the representative histograms describing cell cycle profiles of Hep3B cells. The cell cycle of Hep3B cells was significantly arrested in G0/G1 phase by depleting AKR1B10P1. The results are means of three independent experiments ± SD. (*P < .05). E and F, Cell apoptosis was detected by flow cytometry. As shown the representative histograms describing cell apoptosis status in Hep3B cells. The apoptosis rate of Hep3B cells was significantly increased from 8.75% to 32.38 through AKR1B10P1 depletion. The results are means of three independent experiments ± SD. (**P < .01)

AKR1B10P1 depletion was conducted in Hep3B cells, in which AKR1B10P1 presents the highest level among the three HCC cells (Figure 2A). The cell proliferation was significantly impaired by AKR1B10P1 depletion via shRNA transfection (Figure 2B). *P* value was ＜0.05 for day 1～2 and was ＜0.01 for day 2～4.

The flow cytometric analysis indicates a remarkable arrest of cell cycles at G0/G1 phase in Hep3B cells when AKR1B10P1 depleted (Figure 2C and D). The percentage of the cells in G0/G1 phase was increased from 54.34% to 70.09% (*P *＜ 0.01). The S phase and the G2/M phase were decreased, respectively, from 27.73% to 22.16% (*P *＜ 0.01) and 17.91% to 7.75% (*P *＜ 0.01).

### 3.4 AKR1B10P1 depletion induces cell apoptosis and impairs the ability of cell mobility

Cell apoptosis rate was detected by the flow cytometric analysis. In the AKR1B10P1 depleted Hep3B, cells apoptosis rate was significantly increased from 8.75% to 32.38% (*P *＜ 0.01) (Figure 2E and F).

**FIGURE 3:**
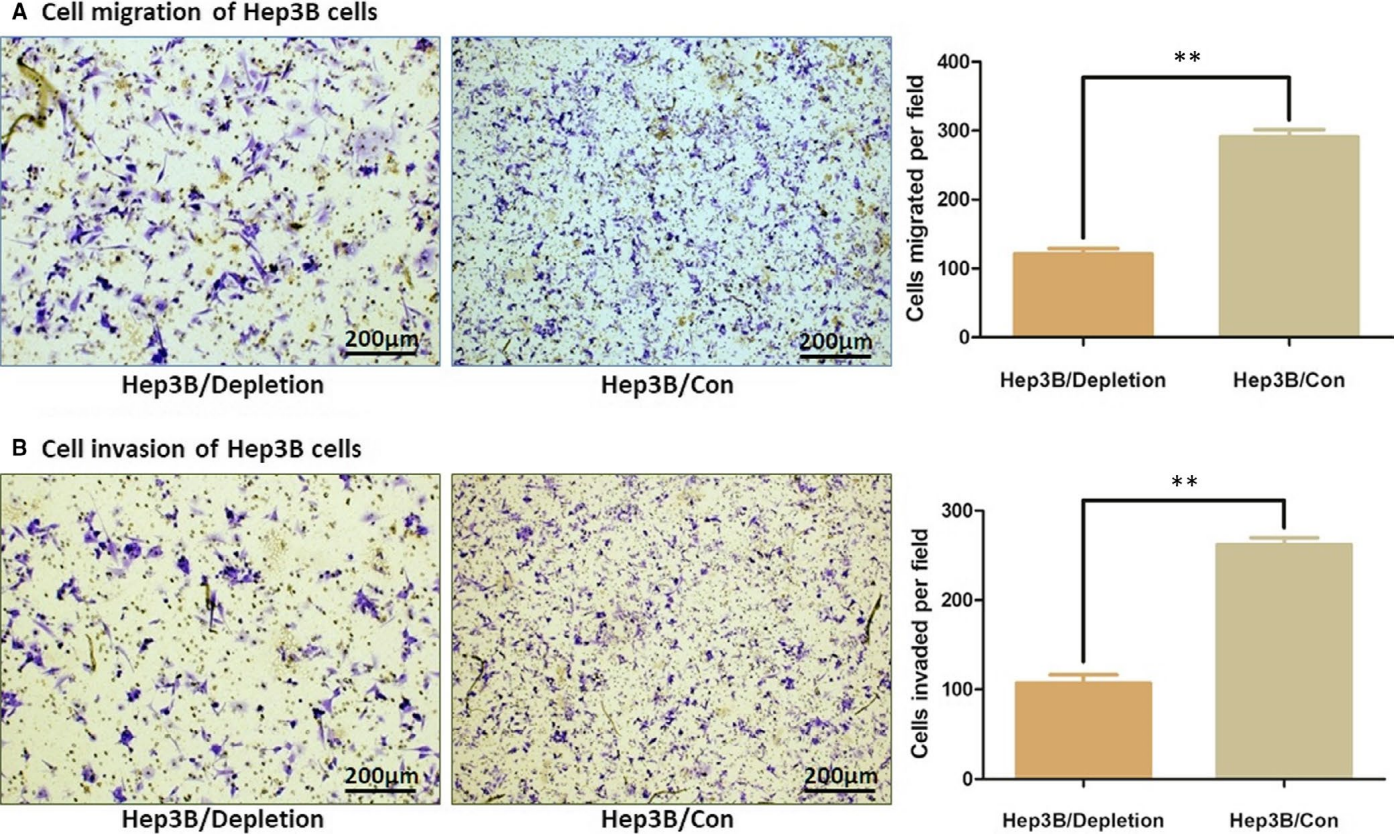
Depletion of AKR1B10P1 impairs the cell motility. Cell migration and invasion of Hep3B cells was detected through the Transwell assay (A) AKR1B10P1 depletion induced a significantly decreased number of Hep3B cells migrated into the low chamber (**P < .01) (B) AKR1B10P1 depletion induced a significantly decreased number of Hep3B cells invaded into the low chamber through the ECMatrixTM pre‐coated (**P < .01)

HCC cell motility was suppressed when AKR1B10P1 depleted. The migration assay demonstrated that the number of cells migrated into the low chamber was remarkably decreased in AKR1B10P1‐depleted Hep3B cells (291 ± 18 cells per field for the negative control, and 121 ± 14 cells per field for the Hep3B/depletion cells, *P *＜ 0.01). The invasion assay indicated the similar result that the number of Hep3B cells invaded into the low chamber was decreased by depleting AKR1B10P1 (262 ± 13 per field for the negative control, and 108 ± 17 cells per field for the Hep3B/depletion cells, *P *＜ 0.01) (Figure 3B).

### 3.5 AKR1B10P1 depletion suppresses HCC in *VIVO*

**FIGURE 4:**
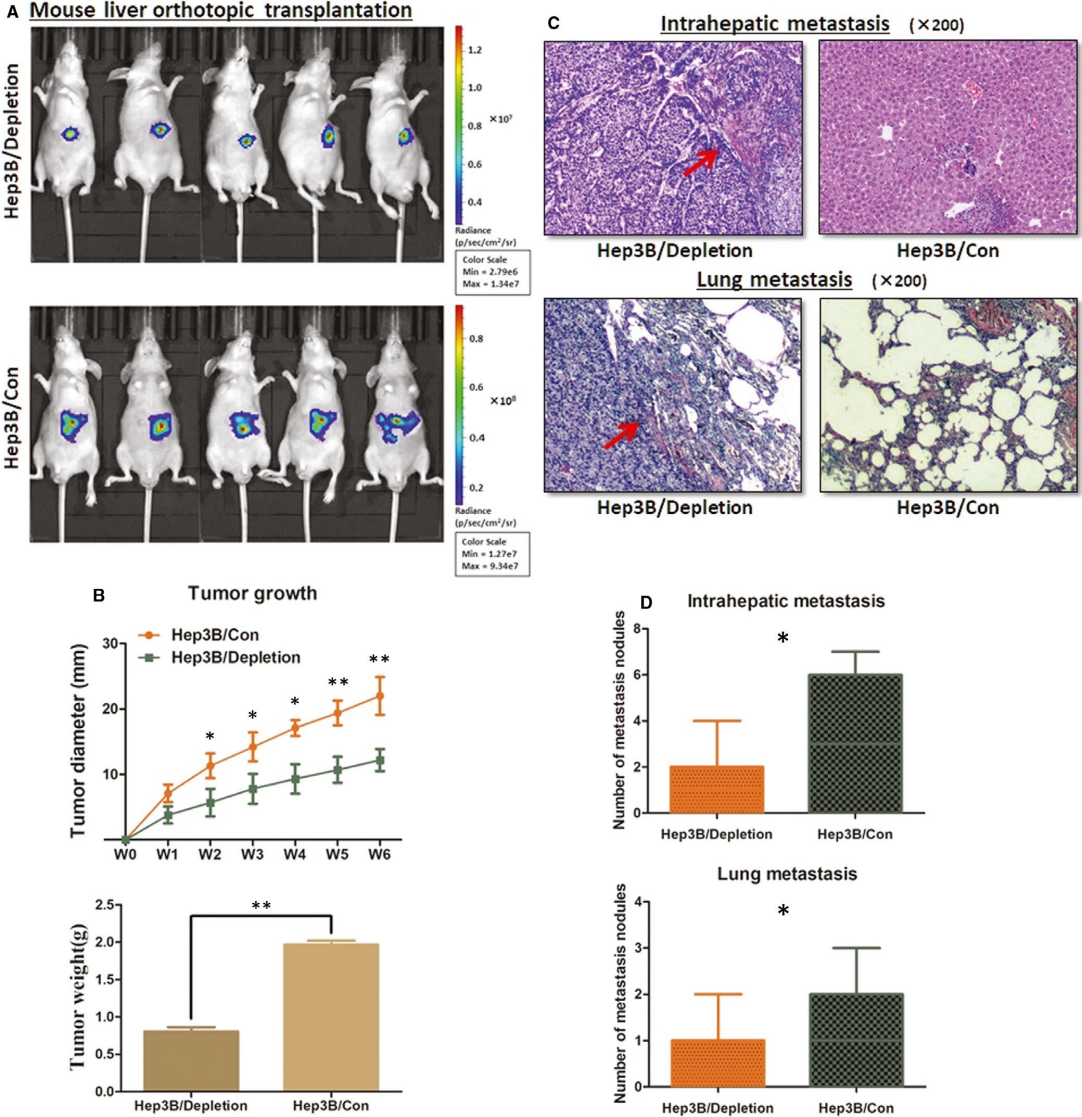
AKR1B10P1 depletion suppresses HCC growth in orthotopic xenograft mouse model. Mouse liver orthotopic transplantation model was constructed in four‐ to five‐week‐old male BALB/c nude mice. A, The orthotopic tumour growth was significantly suppressed in the mice when AKR1B10P1 was depleted in Hep3B cells. B, AKR1B10P1 depletion inhibited the tumour growth and led to the decrease in the xenograft tumour weight in vivo (*P < .01, **P < .01). C and D, The xenograft tumour tissue was checked under HE staining examination. AKR1B10P1‐depleted Hep3B cells induced less intra‐hepatic metastasis lesion and lung metastasis in mice compared with the control Hep3B cells (**P < .01)

Mouse xenograft model was constructed orthotopically to verify the effect of AKR1B10P1 on HCC tumorigenesis in *vivo*. Quantification of the xenografted liver weight 6 weeks later after the injection illustrated that effective AKR1B10P1 depletion in Hep3B cells led to less tumour formation in mice (Figure 4A and B, Figure S3A). HE staining examination was used for further detection the intrahepatic and lung metastatic lesion. The number of micro‐nodules in either liver or lung of the mouse models introduced with AKR1B10P1 depleted Hep3B cells was lower than that of the control group (*P* = .009 for intrahepatic metastasis and *P* = .025 for lung metastasis) (Figure 4C and D).

### 3.6 AKR1B10P1 depletion inhibits EMT activity and depresses SOX4 expression

The process of EMT presents a changeable context of EMT/MET transition in different malignancies, and the tumour cells present hybrid E/M phenotype for involving in multiple steps of the metastatic cascades.^26^ According to the latest guideline on EMT, this process is not a simply binary process.^27^

**FIGURE 5:**
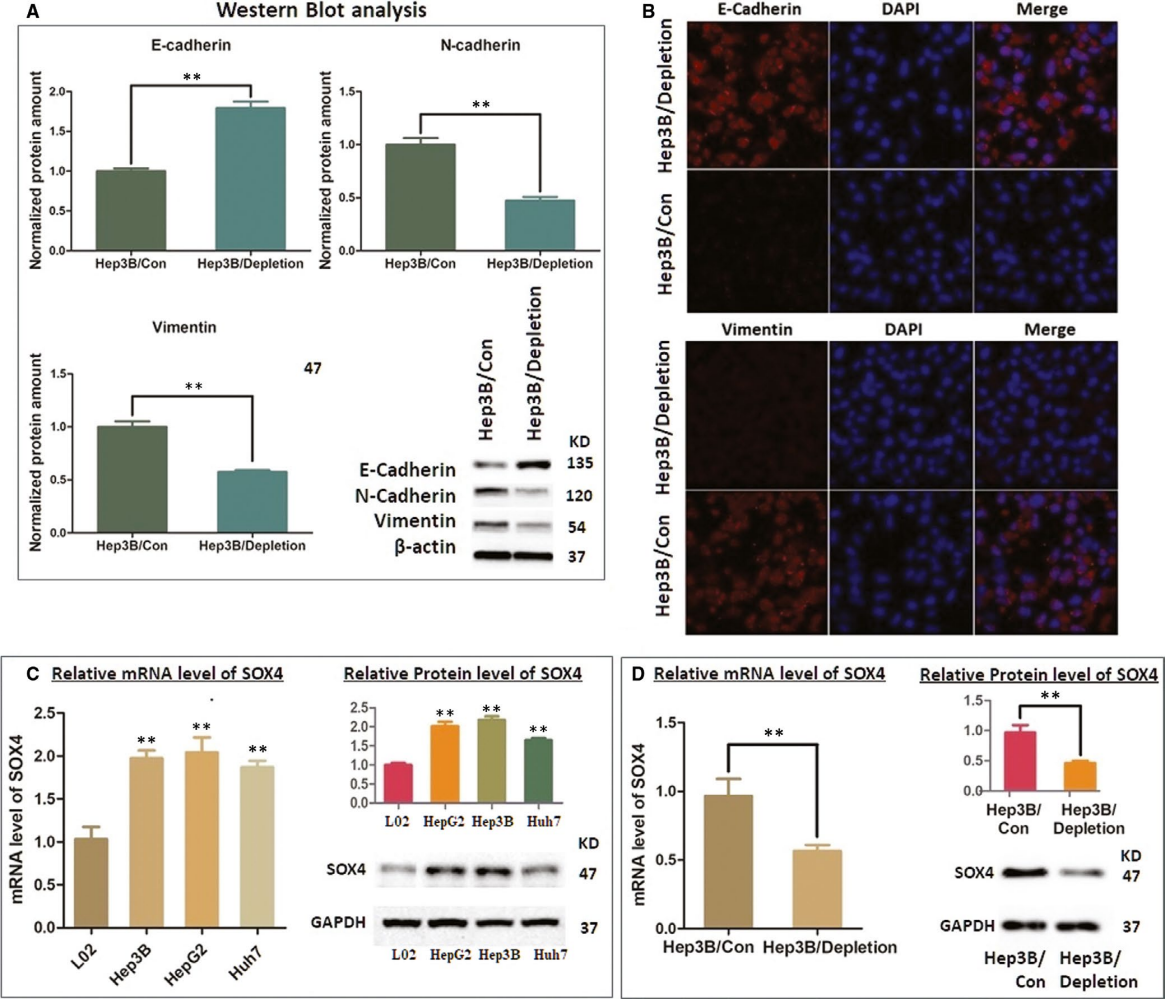
AKR1B10P1 depletion inhibits EMT activity and depresses SOX4 expression in Hep3B cells (A and B) The Western blot analysis and the immunofluorescence assay were used to detect the expression level of EMT indicators. E‐cadherin expression was significantly increased through AKR1B10P1 depletion, compared with the decrease in both N‐cadherin and vimentin (**P < .01). C, Detection of the transcription and protein level of SOX4 in HCC cell lines through RT‐qPCT assay and the Western blot analysis. SOX4 is remarkably up‐regulated in multiple HCC cell lines at both mRNA (**P < .01) and protein status (**P < .01). D. RT‐qPCR assay and the Western blot analysis indicated that by depleting AKR1B10P1 in Hep3B cells, the mRNA and protein expression was significantly reduced (**P < .01)

In this study, we applied the immunofluorescence assay and the Western blot analysis to detect the changes in EMT indicators induced by AKR1B10P1. As we discovered, AKR1B10P1 depletion resulted in a significant up‐regulation of E‐cadherin and an companied decrease in N‐cadherin and vimentin (Figure 5A and B). Although these findings were limited in demonstrating the hybrid E/M cell phenotype, the inhibition of EMT process by depleting AKR1B10p1 was preliminarily observed. And we deduced that the detection focusing on the illustration of the heterogeneity and extent of co‐expression of E/M cells is necessary for intensive study.

SOX4 gene is frequently amplified in more than 20 malignancies, including HCC, and has been reported as one of the master regulators promoting EMT process.^28^, ^29^ In our study, SOX4 was validated up‐regulated in HCC cell lines at both mRNA and protein stage (Figure 5C). Intriguingly, by depleting AKR1B10P1 in Hep3B cells, we observed a significant decrease in SOX4 expression (Figure 5D).

### 3.7 Ectopic expression of SOX4 rescued the EMT‐related indicator changes in AKR1B10P1 depletion

SOX4 expression had been suppressed through AKR1B10P1 depletion according to the examination both in *vivo* and in *vitro* (Figure S3B and C). Given the function of SOX4 in EMT process, we ectopically re‐introduced SOX4 into Hep3B cells treated by AKR1B10P1 depletion with lentiviral vectors.

**FIGURE 6:**
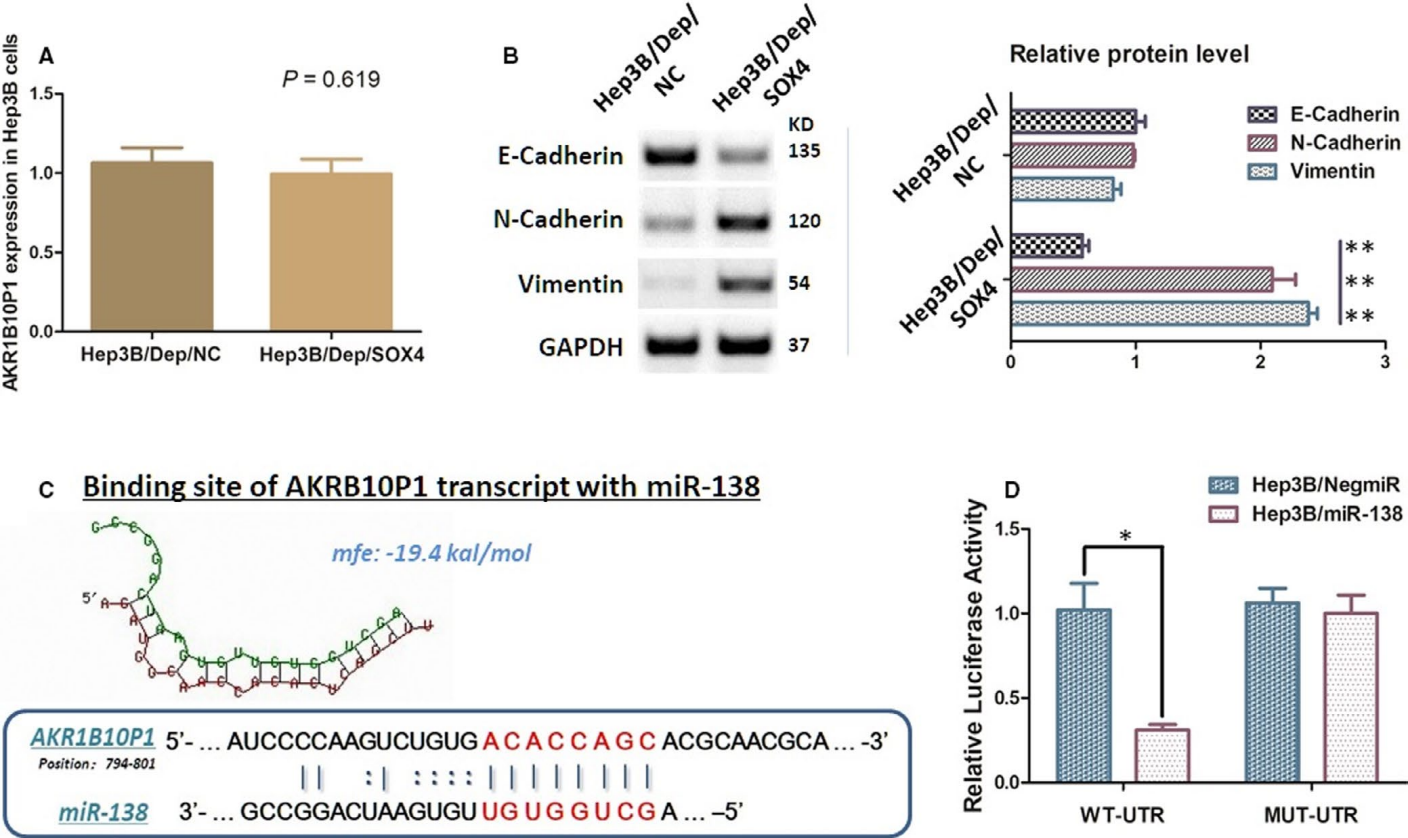
AKR1B10P1 promotes EMT through stabilizing SOX4 mRNA via directly binding with miR‐138 A, RT‐qPCR assay demonstrated that ectopic SOX4 in Hep3B has no significant effect on the expression of AKR1B10, which supports AKR1B10P1 as an up‐streaming regulator of SOX4 (*P* ＞ 0.05). B, The Western blot analysis illustrated that re‐introducing SOX4 ectopically significant rescued the EMT indicator changes induced by AKR1B10P1 depletion. The E‐cadherin protein was decreased, along with the re‐up‐regulation of N‐cadherin and vimentin (*P < .01). C, Predicted binding sequence of AKR1B10P1 transcript with the seed sequence of miR‐138. The minimum free energy (Mfe) hybridization is calculated as follows: −19.4 kal/mol. D, The direct interaction between AKR1B10P1 and miR‐138 was checked by dual‐luciferase reporter assay. Hep3B cells were transfected with the predicted binding site either wild‐type (WT‐binding site) or mutated (MUT‐binding site). MiR‐138 was up‐regulated in these Hep3B cells (Hep3B/miR‐138) through mimics. Up‐regulation of miR‐138 significantly reduced the luciferase signal of the Hep3B cells of WT‐binding site, compared with the negative control (Hep3B/NigmiR); and also, Hep3B cells of WT‐binding site abolished this suppressive effect (**P ＜ 0.01)

As observed, re‐up‐regulating SOX4 barely altered the expression status of AKR1B101P1 in Hep3B cells (*P* = .619) (Figure 6A), but effectively rescued the expression changes of EMT‐related indicators induced by AKR1B10P1 depletion. The expression of E‐cadherin in Hep3B cells was decreased, and N‐cadherin and vimentin expression was increased (Figure 6B). These findings prompt that AKR1B10P1 participates in EMT process through regulating SOX4.

### 3.8 SOX4 is negatively regulated by MIR‐138 IN Hep3B cells

As RT‐qPCR assay demonstrated, miR‐138 expression is relatively lower in HCC cell lines compared with the control L02 cells (Figure S4A). Using microcosm online prediction software (https://www.microcosm.com/), miR‐138 was predicted as an up‐streaming post‐transcriptional regulator of SOX4 (Figure S4B).

MiR‐138 mimicking was introduced into Hep3B cells, and the dual‐luciferase reporter assay was carried out. The vectors containing a 202 bp 3'‐UTR sequence of SOX4 mRNA (WT‐UTR) and the control luciferase vectors containing a mutated miR‐138 target site (MUT‐UTR) were constructed. MiR‐138 significantly declined the luciferase signal of SOX4/pMIR/WT in the Hep3B cells (Hep3B/miR‐138), compared with the control cells (Hep3B/NigmiR). The signal suppressive effect was abolished in Hep3B cells transfected with mutated miR‐138 binding site. All of the above suggest that miR‐138 directly suppresses SOX4 post‐transcriptionally. (Figure S4C).

### 3.9 AKR1B10P1 protects SOX4 mRNA through a ceRNA method binding with miR‐138

We found that there is a sequence within the site from 780bp to 802bp to the 3’ end of the AKR1B10P1 transcript is probably binding to miR‐138, which prompts a ceRNA effect of AKR1B10P1 sponging miR‐138 in HCC cells. The dual‐luciferase reporter assay illustrated that the luciferase signal in Hep3B/miR‐138 cells was significantly decreased when the cells were transfected with AKR1B10P1/pMIR/WT vector (Figure 6C). And the AKR1B10P1/pMIR/MUT vector transfection did not induce significant signal changes (Figure 6D). All these implied us a convincible ceRNA effect of ARK1B10P1 on miR‐138.

## 4 DISCUSSION

The strong invasiveness and motility of HCC cells lead up to 70% of the HCC patients suffered recurrence or metastasis after radical surgery.^30^, ^31^ However, the intensive mechanism intracellular promoting HCC leaves us largely unknown.

Pseudogenes were initiated considered as non‐functional ‘junk DNAs’.^32^ However, current studies address the existence of pseudogene‐derived transcripts and reveal the importance of pseudogenes in human cancers.^33^ The ceRNA effect is one of the most acknowledged functions of pseudogenes and has been validated in different cancers.^34^, ^35^ This provides us a new breakthrough for exploring the complex mechanism of tumorigenesis.

Some of the pseudogenes have been uncovered exerting ceRNA effects on various tumour processes via their transcripts. For instance, glucosylceramidase beta (GBA) is a promotor in gastric cancer targeted by miR‐212‐3p; its pseudogene GBAP1 is aberrantly transcribed in gastric cancer, and maintains GAB expression via sponging miR‐212‐3p.^36^

As for HCC, even though limited in literatures, some pseudogenes have been reported affecting tumour process. For example, the covalently closed circular DNA (cccRNA) is a pivotal factor in HBV‐related HCC by inducing HBV viral persistence, and recently Zhang et.al ^37^ found that the proliferating cell nuclear antigen (PCNA) gene, targeted by miR‐153, is participating in the axis of cccRNA/HBV, which enhance the hepatocarcinogenesis. Meanwhile, PCNA pseudogene transcript (PCNAP1) plays the a role of ceRNA conserving the expression of PCNA by sponging miR‐154. Similarly, onco‐miR‐17‐5p promotes HCC cell growth through degenerating integrator complex subunit 6 pseudogene (INTS6), and up‐regulation of INTS6 pseudogene INTS6P1 could competitively bind to miR‐17‐5p and plays a tumour suppressive role in accordance with INTS6.^38^

In our previous research, AKR1B10 was found as an enhancer of HCC cell growth. Upon the intensive exploration for effect of AKR1B10, we advertently noticed its pseudogene ARK1B10P1 was transcribed in HCC.

AKR1B10P1 gene (chr10:67750284‐67751225) is a processed pseudogene originated from mRNA retro‐transposition, lacking introns. According to the results from the Dataset of dreamBase analysis, AKR1B10P1 transcript is barely detectable in most of the human tissues or organs, including hepatocytes. However, the RT‐qPCR assay gave out a significant expression of AKR1B10P1 in both HCC tissues and cell lines, which prompts the activation of AKR1B10P1 transcription.

Analysis of the clinicopathologic features indicated that AKR1B10P1 transcription associates with larger HCC tumour diameters, higher serum AFP quantity, advanced TNM stages, higher incidence of liver cirrhosis and tumour microsatellite formation. This strongly indicated the function of AKR1B10P1, reciprocally in promoting HCC process along with its parental gene.

Further findings demonstrate that depletion of AKR1B10P1 in HCC Hep3B cells induced tumour suppression in *vitro*, impacting cell growth and mobility, and inducing cell apoptosis. Orthotopic transplantation in mice further demonstrated dramatic decrease in tumour generation and growth in *vivo*. Moreover, AKR1B10P1 depletion reduced the incidence of intrahepatic and lung metastasis in mice. According to these, we suppose AKR1B10P1 exerts enhancing effect on HCC.

On the basis of the findings above, we noticed a strong promotional effect of AKR1B10P1 on cell motility which contributes to tumour invasion and migration. Significantly, the indicators related to EMT process (N‐cadherin, vimentin, E‐cadherin) were consequentially affected when AKR1B10P1 depressed, and the immunofluorescence assay definitely proved a suppressive effect on EMT in HCC cells through AKR1B10P1 inhibition.

SOX4 is a pivotal inducer of EMT in human malignancies contributing to metastasis, which has been validated in various tumours, such as breast cancer, gastric cancer, colon cancer and liver cancer.^39^, ^40^ Considering the effect of AKR1B10P1 on EMT process in HCC, we further investigated and found SOX4 undoubtedly decreased in AKR1B10P1 depleted HCC cells.

The transcription factor SOX4 is a member of the group C subclass in the SOX family. According to the analysis through the Oncomine database (https://www.oncomine.org), SOX4 shows a significant overexpression in 107 out of 464 unique studies covering more than 20 solid tumours, including HCC, and is functionally involved in the activation of multiple signalling pathways regulating tumorigenesis and metastasis, such as PI3K, Wnt, and TGF‐β pathways.^41^, ^42^ By re‐introducing SOX4 in the HCC cells treated with AKR1B10P1 depletion for a rescue experiment purpose, we observed a remarkably reverse change in the phenotypes induced by AKR1B10P1 modulation. The EMT process was reactivated in accordance with the increase in vimentin and E‐cadherin, which strongly indicate the promotional effect of AKR1B10P1 on EMT and metastasis in HCC through the up‐streaming regulation of SOX4.

To make sure of the mechanism AKR1B10P1 regulating the expression of SOX4, we further investigated the sequence of AKR1B10P1 transcript. We discovered a sequence close to the 3’ end of this pseudogene product, which was verified binding to miR‐138, a regulating miRNAs upstream SOX4. We speculated that AKR1B10P1 transcript exerts the ceRNA effect on SOX4. The interaction between miR‐138 and SOX4 mRNA was then confirmed through the dual‐luciferase report assay by mutating the potential binding site located in the 3’‐UTR of SOX4 mRNA. Meanwhile, another dual‐luciferase report system was constructed by specific mutation on AKR1B10P1 transcript to explore the exact interaction between AKR1B10P1 transcript and miR‐138 seed sequence. Although the short sequence of AKR1B10P1 transcript was different from the binding site of SOX4 mRNA, it exhibited strong activation, like a molecular sponge, interacting with miR‐138. These findings supported the ceRNA effect of AKR1B10P1 on stabilization of SOX4 expression.

In summary, we discovered pseudogene AKR1B10P1 abnormally transcribed in HCC tissues and cells consistent with its parental gene, and confirmed the independent tumour enhancing effects of AKR1B10P1. AKR1B10P1 promotes tumour growth both in *vitro* and in *vivo*, and clearly facilitates EMT and tumour motility through stabilizing the EMT inducer SOX4 via the sponge‐like interaction with miR‐138, while intensive understanding of the effects on EMT process involve in AKR1B10P1 remains further investigation. We are aware that some mechanisms have not been illustrated for further understanding, not limited to the EMT process and tumour cell motility, such as the pseudogene‐RNA or RNA binding protein‐RNA interaction, and requiring intensive studying. Our findings certainly brought us some innovative strategies at a molecular level, for HCC research, as well as clinical diagnosis, prevention and therapeutic treatment.

## CONFLICT OF INTEREST

No potential competing interest was disclosed.

## AUTHORS’ CONTRIBUTION

Fengjie Hao: Writing‐original draft (equal). Xiaochun Fei: Data curation (equal); Supervision (equal). Xinping Ren: Formal analysis (equal); Methodology (equal). Joanna Xi Xiao: Writing‐original draft (supporting). Yongjun Chen: Data curation (equal); Investigation (equal). Junqing Wang: Conceptualization (equal); Data curation (equal); Methodology (equal); Writing‐original draft (equal); Writing‐review & editing (equal). Fengjie Hao and Junqing Wang: Writing of the article; Xinping Ren and Joanna Xi Xiao: data analysis and biomolecular experiments; Xiaochun Fei and Nan Wang: in charge of the pathological experiments and data mining; Yongjun Chen: clinicopathological features collection; Junqing Wang: study design and directed the study.

## ETHICS APPROVAL AND CONSENT TO PARTICIPATE

Informed consent was obtained, and the study was approved by the Ethics Committee of Ruijin Hospital, Shanghai Jiaotong University School of Medicine, in accordance with the Declaration of Helsinki.

## Supporting information

Fig S1‐5

Table S1‐2

## Data Availability

Data are available on request from the authors.

## References

[1] Vilgrain V , Pereira H , Assenat E , et al. Efficacy and safety of selective internal radiotherapy with yttrium‐90 resin microspheres compared with sorafenib in locally advanced and inoperable hepatocellular carcinoma (SARAH): An open‐label randomised controlled phase 3 trial. Lancet Oncol. 2017;18(12):1624‐1636. 29107679 10.1016/S1470-2045(17)30683-6

[2] Sartorius K , Sartorius B , Aldous C , et al. Global and country underestimation of hepatocellular carcinoma (HCC) in 2012 and its implications. Cancer Epidemiol. 2015;39(3):284‐290. 25922178 10.1016/j.canep.2015.04.006

[3] Pinyol R , Montal R , Bassaganyas L , et al. Molecular predictors of prevention of recurrence in HCC with sorafenib as adjuvant treatment and prognostic factors in the phase 3 STORM trial. Gut. 2019;68(6):1065‐1075. 30108162 10.1136/gutjnl-2018-316408 PMC6580745

[4] Cancer genome atlas research network. Electronic address wbe , Cancer genome atlas research N. comprehensive and integrative genomic characterization of hepatocellular carcinoma. Cell 2017;169(7):1327‐1341. e1323. 28622513 10.1016/j.cell.2017.05.046 PMC5680778

[5] Chan JJ , Tay Y . Noncoding RNA:RNA regulatory networks in cancer. Int J Mol Sci. 2018;19(5):1310. 10.3390/ijms19051310 PMC5983611 29702599

[6] Yao RW , Wang Y , Chen LL . Cellular functions of long noncoding RNAs. Nat Cell Biol. 2019;21(5):542‐551. 31048766 10.1038/s41556-019-0311-8

[7] Kim M‐S , Pinto SM , Getnet D , et al. A draft map of the human proteome. Nature. 2014;509(7502):575‐581. 24870542 10.1038/nature13302 PMC4403737

[8] Sasidharan R , Gerstein M . Genomics: Protein fossils live on as RNA. Nature. 2008;453(7196):729‐731. 18528383 10.1038/453729a

[9] Rigoutsos I , Furnari F . Gene‐expression forum: Decoy for microRNAs. Nature. 2010;465(7301):1016‐1017. 20577197 10.1038/4651016a

[10] Wang K , Sun Y , Guo C , et al. Androgen receptor regulates ASS1P3/miR‐34a‐5p/ASS1 signaling to promote renal cell carcinoma cell growth. Cell Death Dis. 2019;10(5):339. 31000693 10.1038/s41419-019-1330-x PMC6472417

[11] Thomson DW , Dinger ME . Endogenous microRNA sponges: evidence and controversy. Nat Rev Genet. 2016;17(5):272‐283. 27040487 10.1038/nrg.2016.20

[12] Poliseno L , Salmena L , Zhang J , et al. A coding‐independent function of gene and pseudogene mRNAs regulates tumour biology. Nature. 2010;465(7301):1033‐1038. 20577206 10.1038/nature09144 PMC3206313

[13] Zhang R , Guo Y , Ma Z , et al. Long non‐coding RNA PTENP1 functions as a ceRNA to modulate PTEN level by decoying miR‐106b and miR‐93 in gastric cancer. Oncotarget. 2017;8(16):26079‐26089. 28212532 10.18632/oncotarget.15317 PMC5432239

[14] Karreth FA , Reschke M , Ruocco A , et al. The BRAF pseudogene functions as a competitive endogenous RNA and induces lymphoma in vivo. Cell. 2015;161(2):319‐332. 25843629 10.1016/j.cell.2015.02.043 PMC6922011

[15] Wang J , Zhou Y , Fei X , et al. Biostatistics mining associated method identifies AKR1B10 enhancing hepatocellular carcinoma cell growth and degenerated by miR‐383‐5p. Sci Rep. 2018;8(1):11094. 30038373 10.1038/s41598-018-29271-3 PMC6056456

[16] Wang X , Luo G , Zhang K , et al. Hypoxic tumor‐derived exosomal miR‐301a mediates M2 macrophage polarization via PTEN/PI3Kgamma to promote pancreatic cancer metastasis. Cancer Res. 2018;78(16):4586‐4598. 29880482 10.1158/0008-5472.CAN-17-3841

[17] Wang J , Hao F , Fei X , et al. SPP1 functions as an enhancer of cell growth in hepatocellular carcinoma targeted by miR‐181c. Am J Transl Res. 2019;11(11):6924‐6937. 31814897 PMC6895505

[18] Lin S , Xu J , Hu S , et al. Combined application of neutrophin‐3 gene and neural stem cells is ameliorative to delay of denervated skeletal muscular atrophy after tibial nerve transection in rats. Cell Transplant. 2011;20(3):381‐390. 20719088 10.3727/096368910X524773

[19] Wang J , Yunyun Z , Wang LU , et al. ABCG2 confers promotion in gastric cancer through modulating downstream CRKL in vitro combining with biostatistics mining. Oncotarget. 2017;8(3):5256‐5267. 28029654 10.18632/oncotarget.14128 PMC5354906

[20] Crowley LC , Marfell BJ , Scott AP , et al. Quantitation of apoptosis and necrosis by annexin V binding, propidium iodide uptake, and flow cytometry. Cold Spring Harbor protocols. 2016;2016(11):57‐58. 10.1101/pdb.prot087288 27803250

[21] Wang J , Chen X , Su L , et al. LAT‐1 functions as a promotor in gastric cancer associated with clinicopathologic features. Biomedicine & pharmacotherapy = Biomedecine & pharmacotherapie. 2013;67(8):693‐699. 23809372 10.1016/j.biopha.2013.05.003

[22] Morton CL , Houghton PJ . Establishment of human tumor xenografts in immunodeficient mice. Nat Protoc. 2007;2(2):247‐250. 17406581 10.1038/nprot.2007.25

[23] Donaldson JG , Immunofluorescence staining. Current protocols in cell biology 2001; Chapter 4: Unit 43. 10.1002/0471143030.cb0403s00 PMC4709840 18228363

[24] Zheng L‐L , Zhou K‐R , Liu S , et al. dreamBase: DNA modification, RNA regulation and protein binding of expressed pseudogenes in human health and disease. Nucleic Acids Res. 2018;46(D1):D85‐D91. 29059382 10.1093/nar/gkx972 PMC5753186

[25] Wang J , Zhou Y , Fei X , et al. ADAM9 functions as a promoter of gastric cancer growth which is negatively and post‐transcriptionally regulated by miR‐126. Oncol Rep. 2017;37(4):2033‐2040. 28260063 10.3892/or.2017.5460

[26] Jolly MK , Somarelli JA , Sheth M , et al. Hybrid epithelial/mesenchymal phenotypes promote metastasis and therapy resistance across carcinomas. Pharmacol Ther. 2019;194:161‐184. 30268772 10.1016/j.pharmthera.2018.09.007

[27] Yang J , Antin P , Berx G , et al. Guidelines and definitions for research on epithelial‐mesenchymal transition. Nat Rev Mol Cell Biol. 2020;21(6):341‐352. 32300252 10.1038/s41580-020-0237-9 PMC7250738

[28] Moreno CS . SOX4: The unappreciated oncogene. Semin Cancer Biol. 2019. 10.1016/j.semcancer.2019.08.027 PMC7043201 31445218

[29] Ruan H , Yang H , Wei H , et al. Overexpression of SOX4 promotes cell migration and invasion of renal cell carcinoma by inducing epithelial‐mesenchymal transition. Int J Oncol. 2017;51(1):336‐346. 28534986 10.3892/ijo.2017.4010

[30] Wong MCS , Jiang JY , Goggins WB , et al. International incidence and mortality trends of liver cancer: a global profile. Sci Rep. 2017;7:45846. 28361988 10.1038/srep45846 PMC5374459

[31] Llovet JM , Zucman‐Rossi J , Pikarsky E , et al. Hepatocellular carcinoma. Nature Reviews Disease Primers. 2016;2:16018. 10.1038/nrdp.2016.18 27158749

[32] Sur S , Saha S , Tisa LS , et al. Characterization of pseudogenes in members of the order Frankineae. J Biosci. 2013;38(4):727‐732. 24287652 10.1007/s12038-013-9356-1

[33] Zhu Y , Orre LM , Johansson HJ , et al. Discovery of coding regions in the human genome by integrated proteogenomics analysis workflow. Nat Commun. 2018;9(1):903. 29500430 10.1038/s41467-018-03311-y PMC5834625

[34] Xie C , Zhang L‐Z , Chen Z‐L , et al. A hMTR4‐PDIA3P1‐miR‐125/124‐TRAF6 regulatory axis and its function in NF kappa B signaling and chemoresistance. Hepatology. 2019;71(5):1660‐1677. 31509261 10.1002/hep.30931 PMC7318625

[35] An Y , Furber KL , Ji S . Pseudogenes regulate parental gene expression via ceRNA network. J Cell Mol Med. 2017;21(1):185‐192. 27561207 10.1111/jcmm.12952 PMC5192809

[36] Ma G , Liu H , Du M , et al. A genetic variation in the CpG island of pseudogene GBAP1 promoter is associated with gastric cancer susceptibility. Cancer. 2019;125(14):2465‐2473. 30951202 10.1002/cncr.32081

[37] Feng J , Yang G , Liu Y , et al. LncRNA PCNAP1 modulates hepatitis B virus replication and enhances tumor growth of liver cancer. Theranostics. 2019;9(18):5227‐5245. 31410212 10.7150/thno.34273 PMC6691589

[38] Peng H , Ishida M , Li L , et al. Pseudogene INTS6P1 regulates its cognate gene INTS6 through competitive binding of miR‐17‐5p in hepatocellular carcinoma. Oncotarget. 2015;6(8):5666‐5677. 25686840 10.18632/oncotarget.3290 PMC4467393

[39] Chen J , Ju HL , Yuan XY , et al. SOX4 is a potential prognostic factor in human cancers: a systematic review and meta‐analysis. Clin Transl Oncol. 2016;18(1):65‐72. 26250764 10.1007/s12094-015-1337-4

[40] Li Y , Chen G , Yan Y , et al. CASC15 promotes epithelial to mesenchymal transition and facilitates malignancy of hepatocellular carcinoma cells by increasing TWIST1 gene expression via miR‐33a‐5p sponging. Eur J Pharmacol. 2019;860:172589. 31401158 10.1016/j.ejphar.2019.172589

[41] You S , Knudsen BS , Erho N , et al. Integrated classification of prostate cancer reveals a novel luminal subtype with poor outcome. Cancer Res. 2016;76(17):4948‐4958. 27302169 10.1158/0008-5472.CAN-16-0902 PMC5047668

[42] Grimm D , Bauer J , Wise P , et al. The role of SOX family members in solid tumours and metastasis. Semin Cancer Biol. 2019; S1044‐579X(18)30141‐X. 10.1016/j.semcancer.2019.03.004. [Epub ahead of print] 30914279

