# Supplementary material for: Pseudogene AKR1B10P1 enhances tumorigenicity and regulates epithelial‐mesenchymal transition in hepatocellular carcinoma via stabilizing SOX4
Source: J Cell Mol Med. 2020 Sep 13;24(20):11779–90. doi: 10.1111/jcmm.15790 (PMC7579691; doi:10.1111/jcmm.15790)
Supplement: Supplementary file 1 — Fig S1‐5 [file JCMM-24-11779-s001.docx]

**Supplementary Figures**


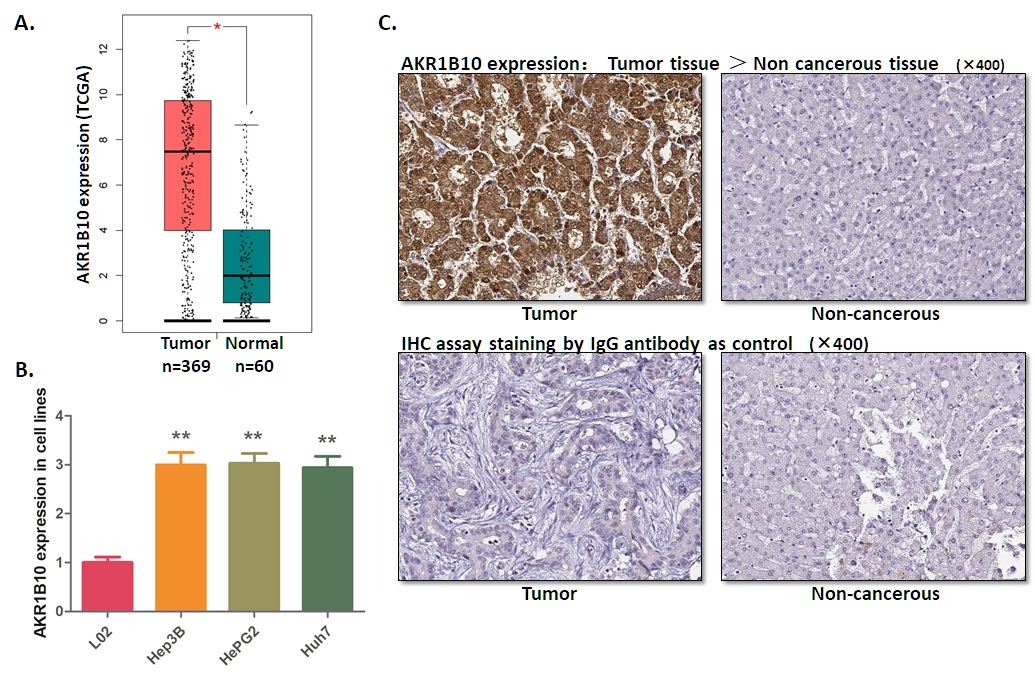


**Suppl. Fig.1. Parental gene AKR1B10 exprssion profile**

**A.** AKR1B10 expression was significantly up-regulated in HCC tissues compared with the normal liver tissues, according to the analysis of TCGA liver cancer datasets (N=429). **B.** AKR1B10 is highly expressed in multiple HCC cell lines compared with the control L02 cells, according to the result of RT-qPCT assay (*P*＜0.01). **C.** Representative graph of immunohistochemistry analysis (400🞩) of the HCC cases. Specimens stained IgG anti-body were regarded as control. AKR1B10 expression in tumor specimens was significantly higher than in adjacent non-cancerous tissues. **
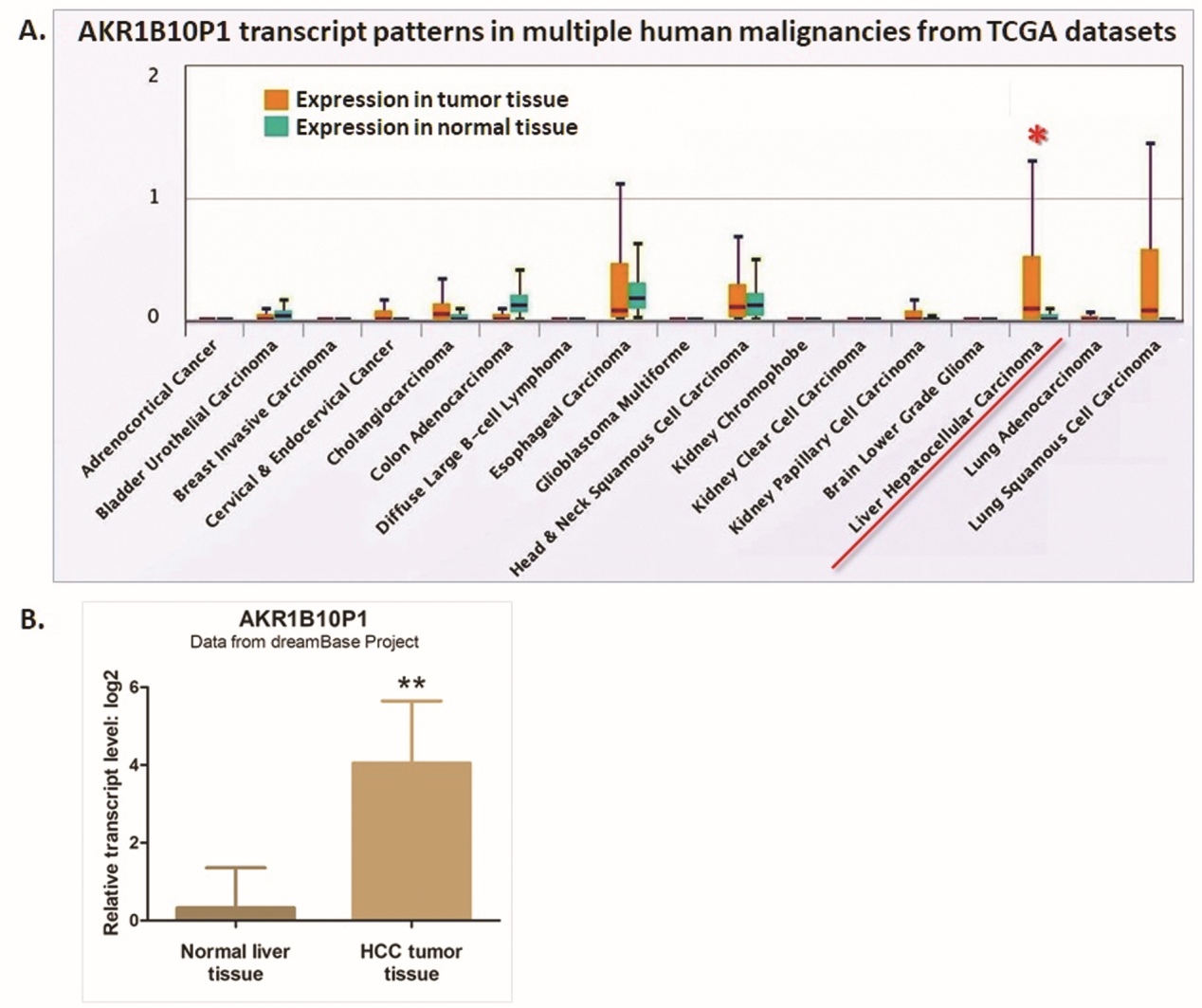
**

**Suppl. Fig.2. Transcription profile of pseudogene AKR1B10P1**

**A.** Histogram of the transcript profile of pseudogene AKR1B10P in multiple human manlignancies. AKR1B10P1 is highly expressed in liver hepatocellular carcinoma tissues, and barely no expressed in normal liver tissues. **B.** Analysis of the dataset from dreamBase Project. AKR1B10P1 was remarkably transcribed in HCC tissues compared with normal liver tissues (***P*＜0.01). **C.** Xenograft tumors were collected and exammed. And we obseverd that SOX4 mRNA exprssion in these tissues were positively correlated with AKR1B10P1 expression (Spearman R=0.7569, *P*=1.1e-3).


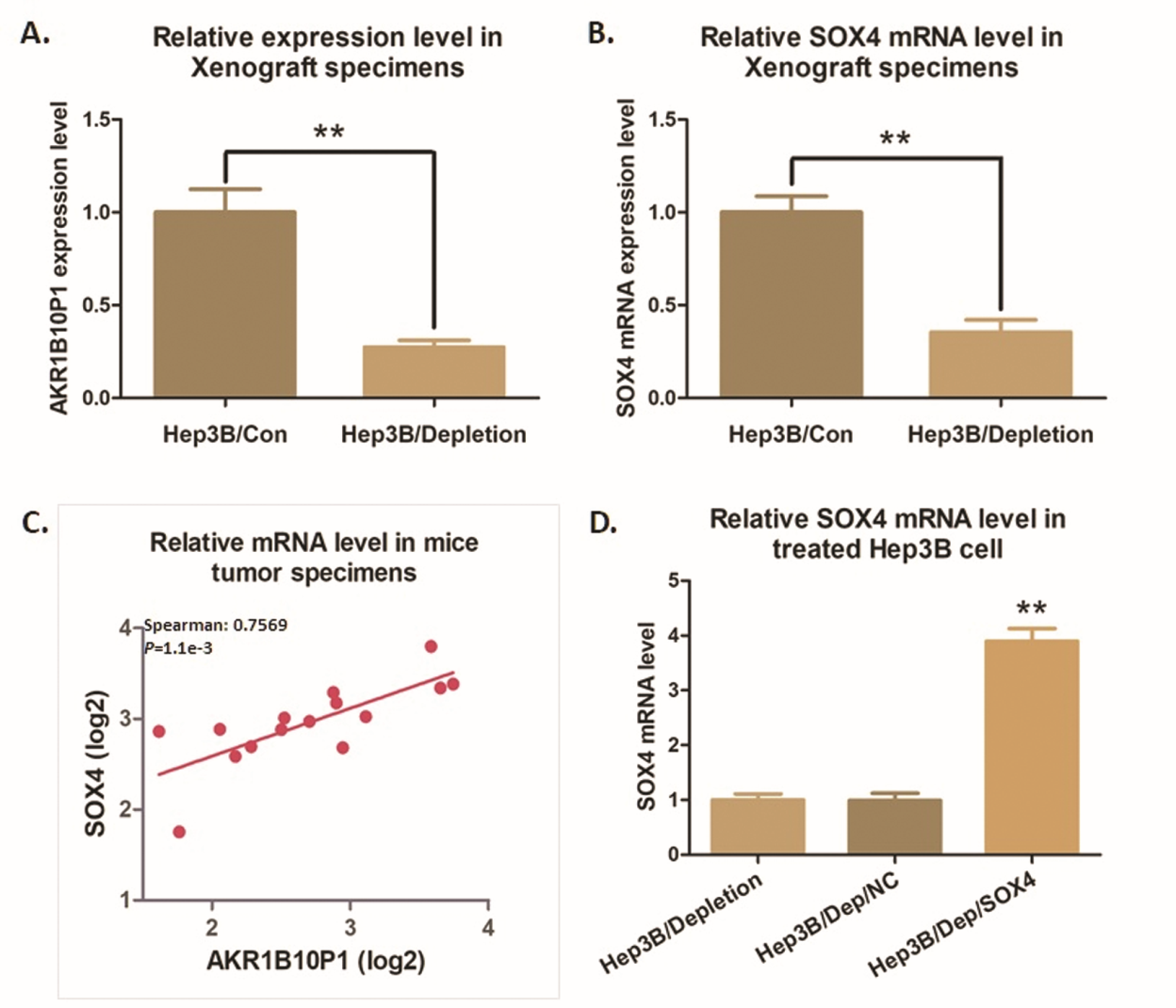


**Suppl. Fig. 3. AKR1B10P1 and SOX4 expression profiles in xenograft mice specimens and cells**

Xenograft tumors were collected and exammed. **A.** Tumors induced by AKR1B10P1 depleted Hep3B cells expressed relatively lower AKR1B10P1 expression compared with the tumor tissues induced by the control cells (***P*＜0.01). **B.** SOX4 mRNA level was relatively lower in the xenograft tumor tissues of AKR1B10P1 depletion compared with the control ones (***P*＜0.01). **C.** In the xenograft tumors, SOX4 mRNA exprssion was positively correlated with AKR1B10P1 expression (Spearman R=0.7569, *P*=1.1e-3). **D.** RT-qPCR assay validated the effectiveness of re-introducing SOX4 via the lentiviral vector pWPXL method in Hep3B cells (***P*＜0.01).


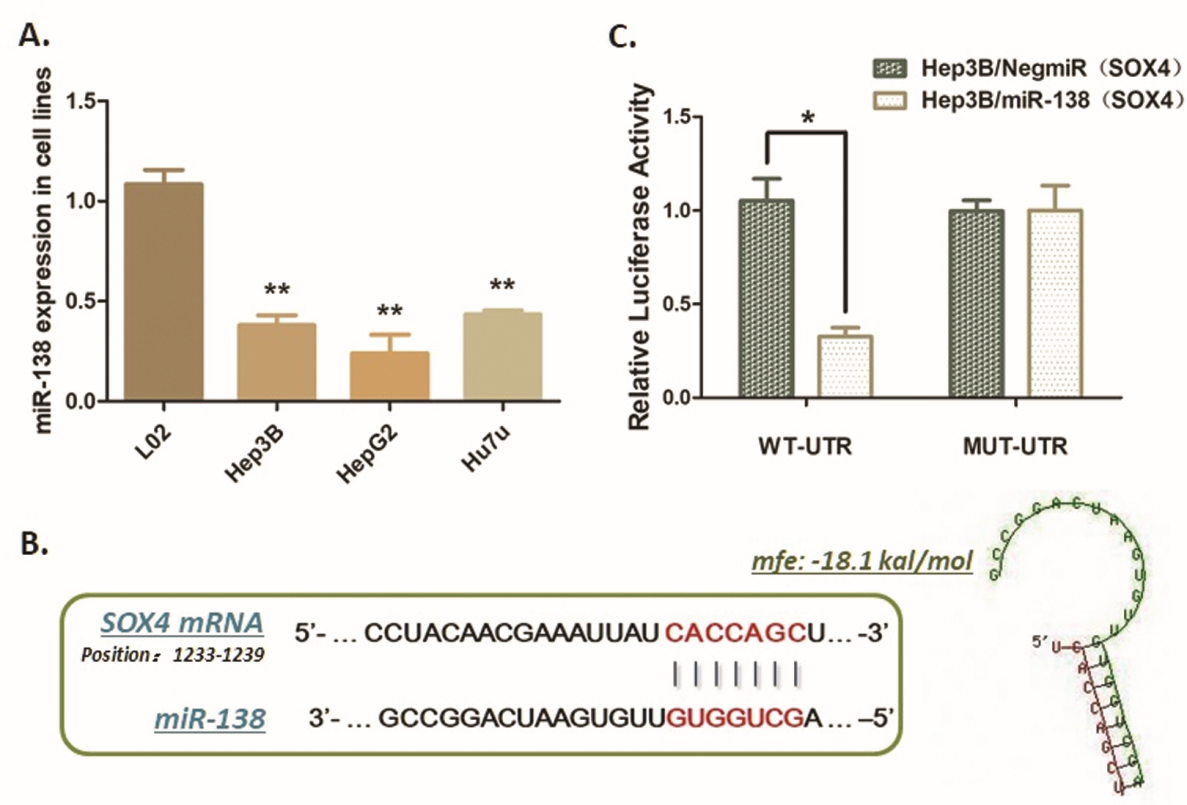


**Suppl. Fig. 4. Regulation of SOX4 in Hep3B cells by miR-138**

**A.** RT-qPCR assay indicated that miR-138 was down-regulated in multiple HCC cell lines (***P*＜0.01). **B.** The predicted miR-138 binding site in the wild type SOX4 mRNA 3’-UTR (WT-UTR) (correspondingly, the mutant construct, defined MUT-UTR, was set. The minimum free energy (Mfe) hybridization is calculated as: -18.1kal/mol. **C.** The direct interaction between miR-138 and SOX4 was checked by Dual-luciferase reporter assay. Up-regulation miR-138 through mimics in Hep3B (Hep3B/miR-138) decreased the luciferase signal of SOX4/pMIR/WT significantly in cells with compared with the negative control (Hep3B/NigmiR) , while mutation of the putative miR-138-binding site abolished this suppressive effect (**P*＜0.05).


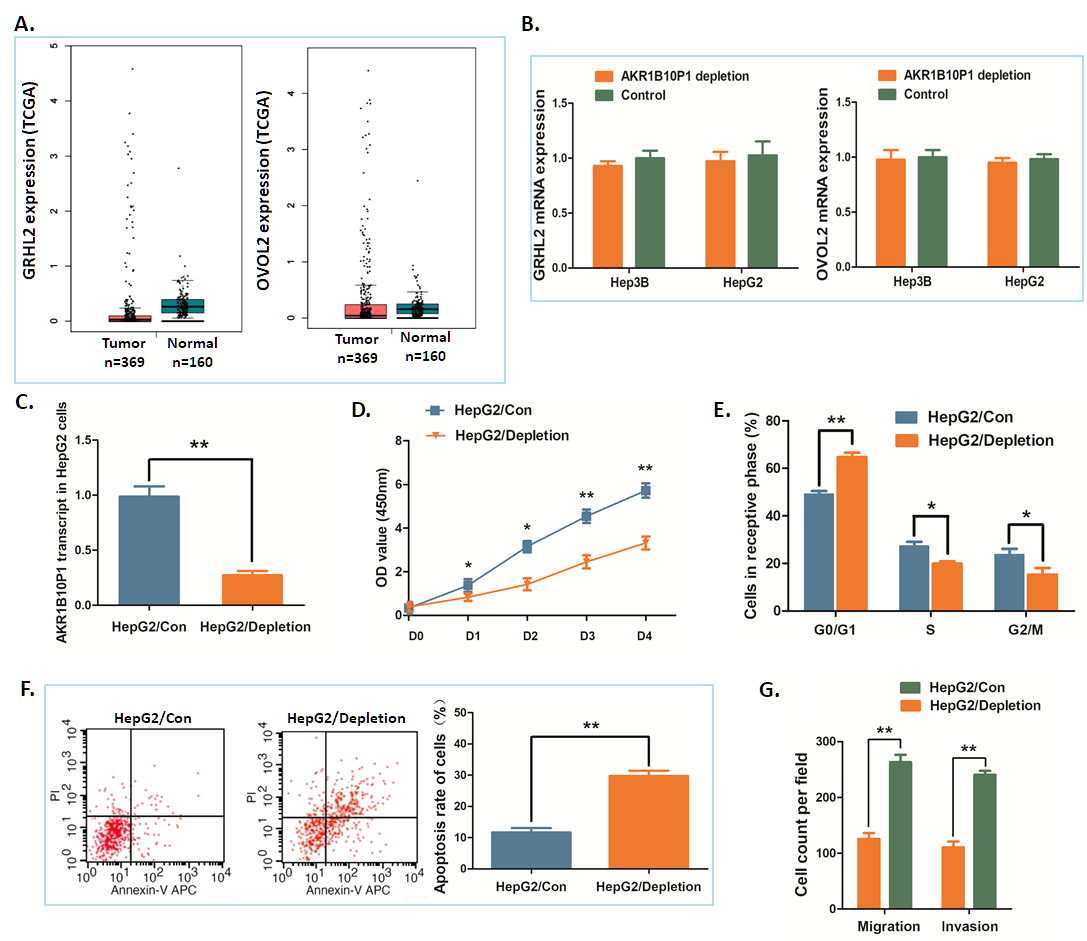


**Suppl. Fig. 5. Expression of EMT indicators in HCC cells and the impact of AKR1B10P1 on HepG2 cells in *vitro***

**A.** Both GRHL2 and OVOL2, which are participating the negative regulation of EMT process, are lowly expressed in either HCC tumor tissues or normal liver tissues without significant difference of expression, according to the analysis of TCGA HCC datasets (N=529). **B.** AKR1B10P1 depletion was conducted in two HCC cell lines, Hep3B and HepG2 cells. There was no significant change of GRHL2 and OVOL2 expression observed, which indicates AKR1B10P1 as an innovative regulator in EMT process independently. **C.** Depletion of AKR1B10P1 in HepG2 cells was validated through RT-qPCR (***P*＜0.01). **D.** CCK8 assay was conducted in HepG2 cells. The cell proliferation of HepG2 cells was impaired by depleting AKR1B10P1 (**P*＜0.05, ***P*＜0.01). **E.** Cell cycle of HepG2 cells was arrested at G0/G1 phase after AKR1B10P1 was depleted according to the flow cytometric analysis. The percentage of the cells in G0/G1 phase was increased from 49.08% to 64.74% (***P*＜0.01). The S phase and the G2/M pahse were decreased respectively from 27.26% to 19.93% (**P*＜0.05) and 23.65% to 15.33% (**P*＜0.05). **F.** Cell apoptosis was detected by flow cytometry. The representative histograms describing cell apoptosis status in HepG2 cells were shown. The apoptosis rate of HepG2 cells was increased significantly from 11.69% to 29.76% after depleting AKR1B10P1. The results are means of three independent experiments ±SD. (***P*< 0.01). **G.** Cell migration and invasion of HepG2 cells was detected through the Transwell assay. The left part of the column chart indicates a significant decrease of HepG2 cells migrated into the low chamber (263±14 cells per field for the negative control, and 126±16 cells per field for the HepG2/Depletion cells, ***P*＜0.01); Meanwhile, the right part of the column chart indicates a significant decrease number of HepG2 cells invaded into the low chamber through the ECMatrixTM pre-coated (241±15 per field for the negative control, and 110±13 cells per field for the HepG2/Depletion cells, ***P* < 0.01).
