# Supplementary material for: Pseudogene AKR1B10P1 enhances tumorigenicity and regulates epithelial‐mesenchymal transition in hepatocellular carcinoma via stabilizing SOX4
Source: J Cell Mol Med. 2020 Sep 13;24(20):11779–90. doi: 10.1111/jcmm.15790 (PMC7579691; doi:10.1111/jcmm.15790)
Supplement: Supplementary file 2 — Table S1‐2 [file JCMM-24-11779-s002.docx]

**Supplementary materials：**

**Suppl. Table. 1**

**The main primers for RT-qPCR**

| **Genes** | **Forward** | **Reverse** |
| --- | --- | --- |
| **AKR1B10P1** | **5’-GGTAATATGATCGGTGGAAAAGCA-3’** | **5’-TAGGGGGCTGTAGGCCATAAT-3’** |
| **AKR1B10** | **5’-CACTGGCCACAGGGATTCAA-3’** | **5’-GGGACATGAGTGGAGGTAGTC-3’** |
| **SOX4** | **5’-GCACTAGGACGTCTGCCTTT-3’** | **5’-ACACGGCATATTGCACAGGA-3’** |
| **miR-138** | **5’-AACGGAGCTGGTGTTGTGAATC-3’** | **5’-GTGCAGGGTCCGAGGT-3’** |
|  |  |  |

**Suppl. Table. 2**

**Selected sequence of the predicted miR-138 binding site of AKR1B10P1 transcript and SOX4 mRNA 3’-UTR, along with the relative mutated sequence**

| **Genes** | **Sequence including the binding site**  **(202 bp)** | **Relative mutated sequence** |
| --- | --- | --- |
| **AKR1B10P1**  **transcrpit** | 5’-agattaaggagattgctgcaaggcacaaaaaaccacagcccaggttctgatccatttccatatccagaggaatgtgactgtgatccccaagtctgtgacaccagcacgcattgttgagaacattcaggcctttgactttacattgaatgatgaggagatggcaaccacactcagcttcaacagaaactggagggcccgtaac-3’ | 5’-acaattacgtgttaggtcctacggaaatatatcgagaccgctgctacagttgcttatgcttttgctgtgcattctcagtctcaacgcgatgacagagtgtggtcggcccttagatcacatcttactgccgtatcagtattcttagtaagttcacgtgttcggatcgagagtgaccatgatctgtatcagcacgccgccttag-3’ |
| **3’-UTR of**  **SOX4 mRNA** | 5’-agatttctgtataagactgttgagcagtttttaaaatagtgtaggataatataaaaagcagatagatggcgctatgtttgattcctacaacgaaattatcaccagctttttttcattcttaactctttaaaggattcaaacgcaactcaaatctgtgctggactttaaaaaaacaattcaggaccaaattttttctcagtgtgtg -3’ | 5’-acaatacagtaaatgtctgatcacctgatataatatttgagaacgtttaaaaatatacctgtttgttcggggtttctatcaatgcaagatccataatttgtggtcgtatatatgaatgtaatcacataatacgttactatccctagtgataacagaggtcgtcataatatatagattactgcagctattatatacactgagagag -3’ |
|  |  |  |
